# Role of Concentrated Growth Factor on the Healing Outcome of Periapical Surgery: A Case Report

**DOI:** 10.7759/cureus.70917

**Published:** 2024-10-06

**Authors:** Alpa Gupta, Dax Abraham, Vivek Aggarwal, Shakila Mahesh

**Affiliations:** 1 Conservative Dentistry and Endodontics, Manav Rachna Dental College, Manav Rachna International Institute of Research and Studies, Faridabad, IND; 2 Conservative Dentistry and Endodontics, Faculty of Dentistry, Jamia Millia Islamia, New Delhi, IND; 3 Microbiology, Manav Rachna Dental College, Manav Rachna International Institute of Research and Studies, Faridabad, IND

**Keywords:** concentrated growth factor, endodontic micro-surgery, periapical surgery, platelet-rich fibrin, platelet-rich plasma

## Abstract

This case report highlights the use of concentrated growth factor (CGF) in enhancing healing outcomes following endodontic periapical surgery. A 57-year-old female came with pain and swelling related to chronic periapical abscesses in her lower front teeth, necessitating surgical intervention. Apicectomy combined with CGF application was considered as a treatment option. CGF, an advanced autologous platelet concentrate, offers superior healing properties due to its natural composition and absence of anticoagulants, making it a favorable option over earlier techniques like platelet-rich plasma (PRP) and platelet-rich fibrin (PRF). The surgical procedure, performed under an operating microscope, included meticulous debridement and the placement of a CGF membrane over the surgical site. Follow-up evaluations at six months and one year demonstrated significant healing, as evident clinically and radiographically. The present case indicated the potential of CGF as an effective adjunct in periapical surgery, promoting better healing and recovery in patients with challenging dental conditions. The findings support the growing interest in autologous biomaterials for regenerative dental procedures.

## Introduction

A surgical approach is used to treat a tooth that has periapical lesions that cannot be treated using an orthograde approach. With the advent of contemporary methods and ideas, positive results have been demonstrated, showing a higher clinical effectiveness following endodontic surgical procedure [1,2]. As a result, regenerative techniques are now recommended as adjuncts in managing such cases. While various regenerative modalities have been proposed, there is growing emphasis on the use of host-derived blood products, which helps in bone healing mainly in oral surgeries [3,4]. The first documented use of platelet-rich fibrin (PRF) was reported by Choukroun et al. PRF is considered superior over conventional platelet-rich plasma (PRP), due to its user-friendliness and entirely autologous nature, as it requires no biochemical processing of blood [5]. Various literature has explored the use of PRP and PRF as healing agents in periapical surgery [4,6]. Concentrated growth factor (CGF) is the newer platelet-derived host material [7]. Unlike PRP, CGF does not contain anticoagulants or bovine thrombin, eliminating the risk of immunological rejection and cross-infection [8]. By accelerating the centrifugation process, which turns fibrinogen into fibrin, it was created from PRF. This could increase the tensile strength of the matrix and encourage the release of platelets and growth factors [8-10].

In terms of composition and processing, CGF performs better than PRP and PRF as a biomaterial [8,9]. It is due to its autogenous origin, clinical use, and biodegradability that CGF has become the choice of material in oral surgical procedures for hard and soft tissue healing [11].

CGF also has immune cells that help control inflammation and lower the risk of infection, as well as higher adhesive strength, tensile strength, and viscosity, all of which support the regeneration process [12]. This case report highlights the application of a host-derived scaffold, utilizing CGF as a healing agent in periapical endodontic surgery.

## Case presentation

**Figure 1:**
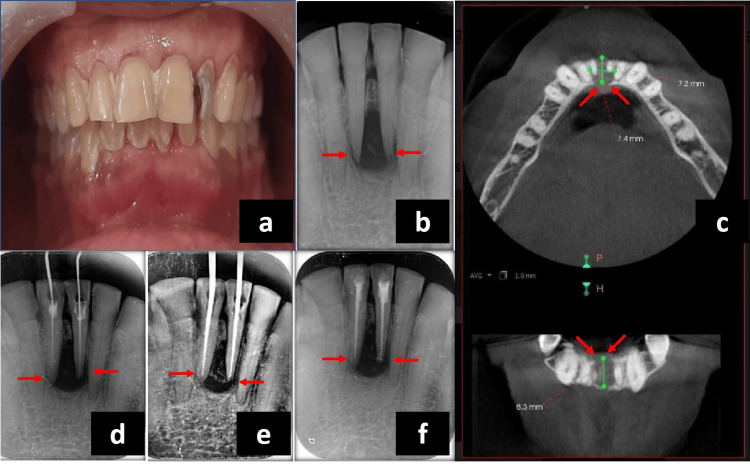
Pre-operative examination (a) Pre-operative intra-oral clinical picture. (b) Pre-operative intra-oral periapical radiograph showing large periapical radiolucency in the mandibular anterior region w.r.t. teeth 31 and 41. (c) Pre-operative cone-beam computed tomography images showing periapical radiolucency with loss of cortical plate on the lingual side in the axial section. (d) Intra-operative working length radiograph. (e) Intra-operative master cone radiograph. (f) Immediate post-obturation radiograph showing satisfactory obturation.

A 57-year-old female patient reported to the specialty clinic with pain and swelling in the lower front tooth region for 2-3 months. The pain was spontaneous in nature and lingering more than 10 seconds after the removal of cold stimulus. The persistent swelling with pus discharge was present in the lower front tooth region. No abnormality was detected on extra-oral examination. Soft tissue examination revealed swelling in the lower front teeth region involving 31 and 41 and intermittent recurring swelling, which was soft in consistency with pus discharge (Figure 1a). Tenderness was positive on percussion w.r.t. 31 and 41. Pulp sensibility testing gave negative responses w.r.t. 31 and 41 indicating non-vital status. Intra-oral periapical radiograph revealed large periapical radiolucency was seen w.r.t. 31 and 41 (Figure 1b). Cone-beam computed tomography (CBCT) was advised (Dentsply Sirona, Orthophos XG 3D, Charlotte, NC, USA) (90kV, 6mA, 5 × 5.5cm, 160μm, and 14secs) to know the size of the lesion (Figure 1c). CBCT revealed that the size of the periapical lesion is more than 6mm and loss of bone at the periapex. Based on the abovementioned findings, a diagnosis of pulpal necrosis with symptomatic apical periodontitis was made. Conventional root canal therapy was planned followed by endodontic surgery w.r.t. 31 and 41 (Figure 1d-1f). It was combined with the incorporation of the CGF membrane in the bony defect. The written consent was obtained from the patient for the surgical procedure. After a proper cleaning and scaling procedure, the surgery was planned.

**Figure 2:**
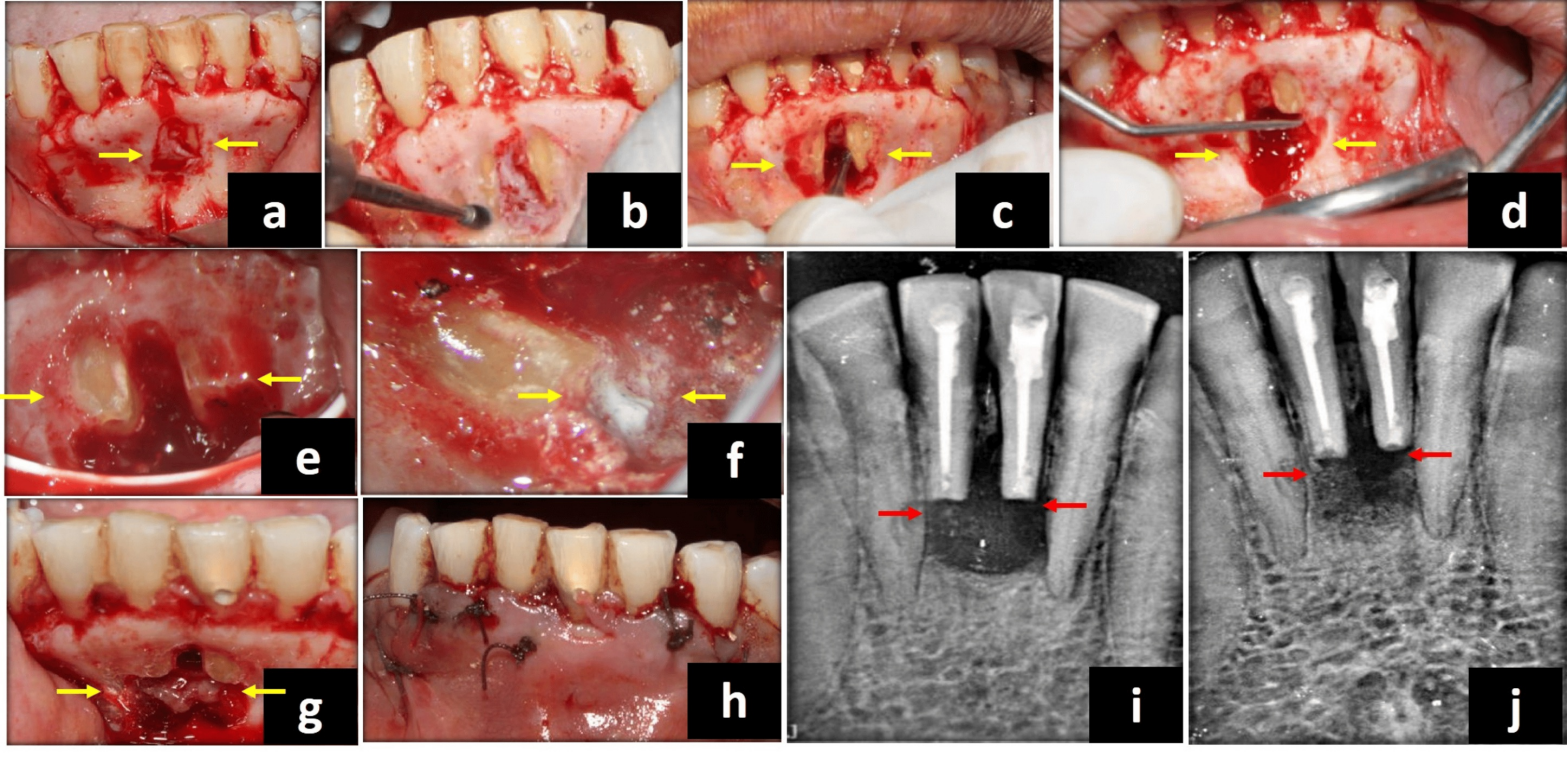
Surgical procedure (a) Full-thickness mucoperiosteal flap elevated between mesial line angle of teeth 33 and 43. (b) Bony window prepared using round carbide bur. (c) Root-end resection of the apical third with a thin straight #701 fissure bur. (d) Use of ultrasonic retro-tips for retro-cavity preparation. (e) Retro-cavity prepared. (f) MTA retro-fill. (g) CGF placement over the lingual perforation site into the bony cavity. (h) Multiple interrupted sutures placed to approximate the flaps. (i) Intra-operative IOPA radiograph to confirm the MTA condensation into canals and no over-fill. (j) Immediate post-surgical IOPA radiograph. MTA: mineral trioxide aggregate; CGF: concentrated growth factor; IOPA: intra-oral periapical

The surgical procedure was performed under a dental operating microscope (Labomed Magna, New York, USA) at 2-24× magnification. Local anesthesia was administered to the patient (Xylocaine; AstraZeneca Pharma, Bangalore, India). With the help of a full-thickness mucoperiosteal flap, the surgical site was exposed, and a bony window was created using a round bur along with constant saline irrigation. The debridement of the surgical site was done (Figure 2a, 2b). A 3mm root resection was done using a #701 carbide bur (SS White Burs, Inc, Lakewood, NJ, USA) along with constant saline irrigation (Figure 2c). The root end was prepared at a depth of 3mm with the help of ultrasonic retro-tips (Satelec, Paris, France) (P5 Booster, Suprasson Newtron; Acteon Inc, Mt Laurel, NJ, USA) at medium power setting (Figure 2d). Finally, the root end was sealed with mineral trioxide aggregate (MTA) (Pro Root; Retroplast Trading, Rorvig, Denmark) (Figure 2e, 2f). 

**Figure 3:**
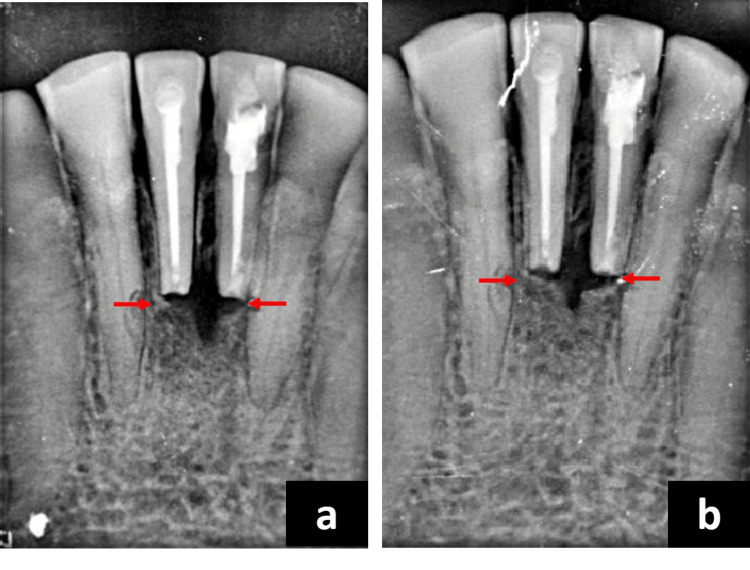
Follow-up radiographs (a) Follow-up IOPA radiograph at six months. (b) Follow-up IOPA radiograph at 12 months shows the progression of healing and bone-fill at the periapical region of 31 and 42. IOPA: intra-oral periapical

Following the patient's written consent, the necessary blood was drawn into a 10ml test tube to prepare the CGF, which was then quickly centrifuged using a tabletop centrifuge (MEDIFUGE CGF, Silfradent, Santa Sofia, Italy). The machine's "preprogramming" allowed for the automatic adjustment of each of these procedures. The final product was segregated into three layers. Using a sterile syringe, the topmost layer which was devoid of platelets was extracted from the three layers that had formed. The CGF-containing fibrin gel layer was isolated. Following that, the CGF membrane was obtained by compressing the CGF layer (Figure 2g). The prepared CGF product was placed over the surgical site at the periapical defect area. Two post-operative radiographs at different angulations were taken after suturing the surgical site (Figure 2h, 2i, 2j). The patient was instructed to take care of proper oral hygiene. Suture removal was done on the fourth day after surgery. The follow-up radiograph was taken at six months and one year (Figure 3a, 3b). 

## Discussion

Recent research aimed towards improving periapical healing, based on increasing focus on autologous platelet concentrates. PRP and PRF have garnered significant attention for their healing properties [13]. Studies have demonstrated that PRP positively promotes both hard and soft tissue healing. PRP has been shown in numerous trials to produce positive results in periodontal and oral and maxillofacial operations [14]. Various research evaluated the effectiveness of these autologous platelet products in surgical procedure [15-18]. We chose CGF due to its multifunctional benefits: it acts as a barrier against epithelial migration. CGF promotes neo-angiogenesis and stabilizes the flap as a fibrin glue. Additionally, the α-granules in CGF platelets are reservoirs of growth factors crucial for healing and repair [19]. When it comes to the amount of growth factor release, CGF releases more than PRF [20]. The entire process was carried out under a microscope to improve the periapical area's accessibility and visibility. Due to its excellent sealing qualities, MTA, the most popular retro-filling material, was chosen. However, more follow-up is required. The present clinical case shows the significant and beneficial application of CGF in facilitating the healing process of extensive periapical lesions located in the anterior region of the mandible. Microendodontic surgical intervention was meticulously carried out. The standard and widely accepted protocol for endodontic microsurgical procedures was diligently adhered to throughout the course of treatment. In the present case that we are discussing, the utilization of CGF resulted in a remarkable and observable degree of healing at both the six-month and one-year follow-up evaluations, which were conducted to assess the outcomes of the treatment. The underlying mechanism that likely accounts for this notable healing effect can be attributed to the substantial release of a considerable quantity of growth factors by the CGF, a phenomenon that appears to be more pronounced when compared to other types of autologous platelet aggregates that are commonly used in clinical practice. It is imperative to conduct a well-designed clinical trial in order to conclusively demonstrate and establish the superior clinical efficacy of CGF in comparison to other available therapeutic modalities. 

## Conclusions

​​Within the scope of the present case, it can be suggested that CGF can be a safe and effective option for healing in periapical surgical cases. Further research with more clinical trials consisting of large sample sizes is required to prove the enhanced clinical efficiency of CGF over other autologous platelet aggregates. 
